# Brief Psychotherapeutic and Psychopharmacological Interventions as Facilitators of Bariatric Surgery Success in Patients on the Anxious-Impulsive Spectrum: A Pilot Study

**DOI:** 10.1192/j.eurpsy.2024.1020

**Published:** 2024-08-27

**Authors:** Í. Alberdi-Páramo, M. Navas Tejedor, M. Paz Otero, J. Sánchez- Rodríguez, D. Gimeno Álvarez

**Affiliations:** ^1^ Hospital Clínico San Carlos; ^2^ Universidad Complutense; ^3^Psiquiatría, Hospital Clínico San Carlos, Madrid, Spain

## Abstract

**Introduction:**

Patients undergoing bariatric surgery often present with impulsive behavior and symptoms of anxiety. In this context, brief psychotherapeutic interventions such as nutritional education, cognitive restructuring, and behavioral activation have been shown to enhance pre-surgery weight loss and improve the likelihood of successful surgical outcomes. Furthermore, anorexigenic pharmacological treatments involving fluoxetine, bupropion, naltrexone, eslicarbazepine, zonisamide, and topiramate have been associated with increased success rates of the bariatric intervention.

**Objectives:**

To assess the impact of brief psychotherapeutic interventions and psychopharmacological treatments on the success of bariatric surgery in anxious-impulsive patients, investigating the effectiveness of combined strategies in enhancing preoperative weight loss and surgical outcomes.

**Methods:**

Within the framework of a third-level hospital’s Bariatric Surgery Protocol, a total of 63 obese patients were assessed using the MINI International Neuropsychiatric Interview (MINI), Hamilton Anxiety Rating Scale (HARS), and Barratt Impulsiveness Scale (BIS-11) during the pre-surgical evaluation. Patients with Axis I pathologies were excluded, leaving a sample of 56 participants (38 females; BMI: 43.58±8.72 kg/m2; age: 48.5±9.7 years). Individuals displaying mild anxiety (6-14 points on HARS) and moderate/severe anxiety (>14 points on HARS) and/or those with a BIS-11 score exceeding 32.5 were selected for combined psychotherapeutic and psychopharmacological interventions.

**Results:**

Categorized by anxiety and impulsiveness levels, the patient distribution was as follows:

Mild anxiety without impulsiveness: 19 patients

Mild anxiety with impulsiveness: 31 patients

Moderate/severe anxiety without impulsiveness: 2 patients

Moderate/severe anxiety with impulsiveness: 15 patients

This pilot study explores the potential synergy between brief psychotherapeutic interventions and psychopharmacological approaches in enhancing the outcomes of bariatric surgery for patients within the anxious-impulsive spectrum.

**Conclusions:**

The results shed light on the feasibility and potential benefits of a combined treatment strategy, contributing to the optimization of bariatric surgery success in this specific patient population. Further research is warranted to confirm and generalize these findings.

**Disclosure of Interest:**

None Declared

